# Hyaluronic Acid (HA) Receptors and the Motility of Schwann Cell(-Like) Phenotypes

**DOI:** 10.3390/cells9061477

**Published:** 2020-06-17

**Authors:** Sihem Ouasti, Alessandro Faroni, Paul J. Kingham, Matilde Ghibaudi, Adam J. Reid, Nicola Tirelli

**Affiliations:** 1 Division of Pharmacy and Optometry, School of Health Sciences, Faculty of Biology Medicine and Health, University of Manchester, Manchester Academic Health Science Centre, Manchester M13 9PL, UK; Sihemouasti@hotmail.com; 2 Blond McIndoe Laboratories, Division of Cell Matrix Biology and Regenerative Medicine, School of Biological Sciences, Faculty of Biology Medicine and Health, University of Manchester, Manchester Academic Health Science Centre, Manchester M13 9PL, UK; alessandro.faroni@manchester.ac.uk (A.F.); paul.kingham@umu.se (P.J.K.); Adam.Reid@manchester.ac.uk (A.J.R.); 3 Department of Integrative Medical Biology, Section of Anatomy, Umeå University, 901 87 Umeå, Sweden; 4 Laboratory of Polymers and Biomaterials, Fondazione Istituto Italiano di Tecnologia, 16163 Genova, Italy; matilde.ghibaudi@iit.it; 5 Department of Plastic Surgery & Burns, Wythenshawe Hospital, Manchester University NHS Foundation Trust, Manchester Academic Health Science Centre, Manchester M23 9LT, UK

**Keywords:** CD44, RHAMM, Schwann cells, motility

## Abstract

The cluster of differentiation 44 (CD44) and the hyaluronan-mediated motility receptor (RHAMM), also known as CD168, are perhaps the most studied receptors for hyaluronic acid (HA); among their various functions, both are known to play a role in the motility of a number of cell types. In peripheral nerve regeneration, the stimulation of glial cell motility has potential to lead to better therapeutic outcomes, thus this study aimed to ascertain the presence of these receptors in Schwann cells (rat adult aSCs and neonatal nSCs) and to confirm their influence on motility. We included also a Schwann-like phenotype (dAD-MSCs) derived from adipose-derived mesenchymal stem cells (uAD-MSCs), as a possible basis for an autologous cell therapy. CD44 was expressed similarly in all cell types. Interestingly, uAD-MSCs were RHAMM(low), whereas both Schwann cells and dASCs turned out to be similarly RHAMM(high), and indeed antibody blockage of RHAMM effectively immobilized (in vitro scratch wound assay) all the RHAMM(high) Schwann(-like) types, but not the RHAMM(low) uAD-MSCs. Blocking CD44, on the other hand, affected considerably more uAD-MSCs than the Schwann(-like) cells, while the combined blockage of the two receptors immobilized all cells. The results therefore indicate that Schwann-like cells have a specifically RHAMM-sensitive motility, where the motility of precursor cells such as uAD-MSCs is CD44- but not RHAMM-sensitive; our data also suggest that CD44 and RHAMM may be using complementary motility-controlling circuits.

## 1. Introduction

The traditional approach to repair an injury of the peripheral nervous system (PNS) is the reconnection of the proximal and distal extremities of the severed nerve, with use of autologous nerve grafting reserved for severe injuries to bridge large defects [1]. In the last twenty years, regenerative therapies have gained experimental popularity [2,3], with the main aim to achieve a therapeutic success at the lesion site without significant donor side co-morbidities, e.g., sensory loss and neuropathic pain at the donor site. In this area, two points can summarize the state of the art: a) a (resorbable) conduit, which guides the regenerative process [4,5,6,7], and b) its combination at the injury site with cells that can enhance the axon re-growth, e.g., Schwann or precursor/stem cells [8,9] (or factors secreted by them [10,11]). Indeed, functional Schwann-like cells can be produced in large numbers through differentiation from low-morbidity precursor cells, which can be derived from adipose tissue [12,13] or a variety of other sources, including bone marrow, skin, or hair follicles [3]. We are specifically interested in adipose-derived mesenchymal stem cells (AD-MSCs), because they are more accessible, require a simpler isolation procedure [14], and have been successfully differentiated into Schwann-like phenotype [15,16] (although less effectively when of human origin [17]); in vivo, they have been shown to stimulate nerve regeneration [18,19,20], but suffer a relatively short survival in the injured environment (<2 weeks in poly(3-hydroxybutyrate) conduits [21]). They have been employed in several non-resorbable (silicone [19]) or resorbable conduits, including fibrin [22], collagen [23], poly(glycolic acid)/collagen [24], however to our knowledge, never in biomimetic environments designed to stimulate their survival and motility.

On the other hand, whether Schwann cells are sourced directly or differentiated from precursors, their efficacy is modulated by their microenvironment, and specifically by their extracellular matrices (ECMs) [25,26]. In particular, the motility of the Schwann(-like) cells is a key parameter for an efficacious peripheral nerve regeneration, and it may be strongly influenced by the interactions with ECMs.

In the PNS, ECMs often lack classical matrix proteins (collagens, fibronectin, and vitronectin), but are rich in hyaluronic acid (HA) and in HA-binding proteins (hyalectans: brevican, neurocan, aggrecan, and versican) [27,28]. HA is an almost ubiquitous glycosaminoglycan with a multiplicity of matrix-associated and signaling roles; they include also mediating cell motility, as it has been well demonstrated in cancer [29,30], typically in combination with its two often cross-talking receptors CD44 and RHAMM [31]. It is worth pointing out that CD44 has a very important—although not yet completely understood [32,33]—role in HA uptake and endocytosis, while RHAMM-HA interactions have never been convincingly related to internalization; their cross-talks, therefore, may occur at the level of intracellular HA trafficking or in their respective signaling cascades.

For what attains to the role of HA and its receptors in the nervous system and specifically in PNS, there is evidence supporting a role in cell motility also here. A few examples: (a) during development; for instance, HA accumulates at sites of neuronal differentiation in the spinal cord (chicken embryos) [34], migrating neural crest cells express large amounts of CD44 (rat embryos) [35], and CD44 expression correlates with that of RHAMM and with migration and proliferation capacity during development (*Xenopus laevis*) [36]; (b) during the inflammatory consequences of an injury, CD44 expression is essential for the migration of re-myelinating oligodendrocyte precursors following spinal injury (rats) [37], and in the sciatic nerve HA is produced by proliferating Schwann cells during Wallerian degeneration (rats) [38]; (c) in tumors, examples include HA being overexpressed in malignant peripheral nerve sheath tumors [39] (although RHAMM is reportedly inversely correlated to their invasiveness [40]), CD44 being a marker of invasiveness for gliomas [41] and specifically for their initiating cells [42], and both CD44 and RHAMM having been shown to mediate glioma migration and proliferation [43].

As a testament of the HA capacity to stimulate cell motility—but also to reduce (perineural) scarring—the administration of soluble HA is beneficial for an accelerated regeneration of peripheral nerves [44,45]; HA can also be fashioned in a variety of hydrogel morphologies, including photopolymerized materials [46,47], and indeed HA-based Schwann cell-seeded matrices have been used within nerve conduits [48].

**Scheme 1:**
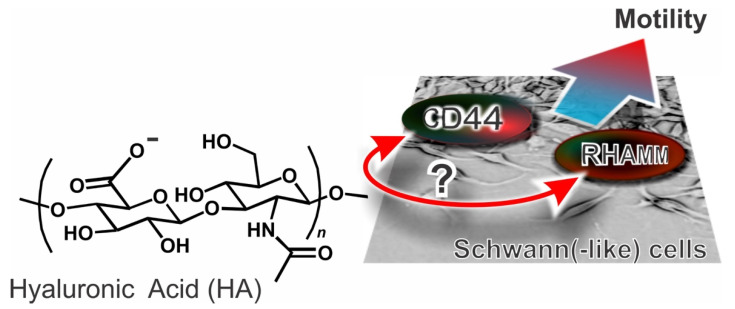
The main questions of this study are whether a) hyaluronic acid-based motility signaling may be equally operational in Schwann cells and in Schwann-like phenotypes and b) both of its main receptors are involved.

The aim of this study is to understand whether (1) a qualitative correlation may be observed between HA, the presence of its receptors and the motility of Schwann cells (Scheme 1), and may entail additive or overlapping effects of CD44 and RHAMM, and (2) this correlation remains valid in a Schwann cell-like phenotype produced from precursor cells harvested from non-PNS sources, namely AD-MSCs. Importantly, as Schwann cell controls we have used both neonatal and adult cells, since the former might bear a higher similarity to the freshly differentiated AD-MSCs.

## 2. Materials and Methods

### 2.1. Experimental Section

Details of the animals (sciatic nerve samples and primary cells were harvested from 3-month old Sprague-Dawley (SD) rats, which were terminated according to schedule 1 procedures of the UK Animals (Scientific Procedures) Act 1986) and materials, and of the methods for histochemical, semi-quantitative reverse-transcriptase PCR (RT-PCR), and immunocytochemical and Western blotting analyses are provided in Supplementary Materials, respectively in Sections S1.1, S1.2, S1.3, S1.4, and S1.5. 

### 2.2. Cell Culture

Neonatal SC were obtained from sciatic nerves P1-2 Sprague Dawley rats using an established protocol [49]. The nerves were aseptically harvested and collected in chilled low glucose Dulbecco’s modified Eagle’s medium (DMEM; Sigma-Aldrich, Gillingham, UK) with 1% (*v/v*) of penicillin-streptomycin solution (P-S, PAA Laboratories, Leeds, UK). Nerves were digested with 1% of collagenase Type I (Worthington Biochemicals, Lakewood, CA, USA), and 0.25% of trypsin- ethylenediaminetetraacetic acid (trypsin/EDTA; Invitrogen, Warrington, UK) for 1 h at 30 °C. The digested nerves were mechanically triturated through a 23-gauge needle and filtered through a 100 μm BD FalconTM cell strainers (BD Bioscience, Wokingham, UK). The resulting cell suspension was centrifuged for 5 min at 900 rpm and plated on a poly(D-lysine) (PDL; Sigma-Aldrich) coated 25 cm^2^ flask at 37 °C and 5% CO_2_. Treatment with 10 μM of cytosine β-D-arabinofuranoside (Ara-C; Sigma-Aldrich) was performed for 24 h to reduce fibroblast contamination. Schwann cells were cultured in DMEM supplemented with 14 μM forskolin (fsk; Sigma-Aldrich) and 63 ng/mL of glial growth factor-2 (GGF-2; Acorda Therapeutics Inc., Waltham, MA, USA). Once the flask was confluent, the SC population was further purified by immunopanning [49].

Adult SC (aSC) were harvested from the sciatic nerves of adult male Sprague-Dawley rats using a previously established protocol [18]. The epineurium was removed and the nerves were cut into 1 mm pieces prior to incubation in DMEM for two weeks with media changes every 3 days. This has been shown to increase Schwann cell yield and reduce fibroblast contamination [50]. Nerve biopsies were then digested with 0.0625% (*w/v*) collagenase type IV (Worthington Biochemicals) and 0.585 mg/mL of dispase (1.17 U/mg; Invitrogen) for 24 h at 37 °C. Finally, the digested nerves were mechanically dissociated, filtered through 100 μm BD Falcon^TM^ cell strainers, plated into PDL coated 75 cm^2^ flasks, and maintained in DMEM supplemented with 14 μM forskolin (fsk; Sigma-Aldrich) and 63 ng/mL of glial growth factor-2 (GGF-2; Acorda Therapeutics Inc.). Confluent flasks were purified by immunopanning to reduce fibroblast contamination.

#### 2.2.1. Undifferentiated Adipose-Derived Mesenchymal Stem Cells (uAD-MSCs)

Fat tissue from the subcutaneous and abdominal region (surrounding stomach and intestine) of SD male rats was carefully removed and mechanically dissociated with a sterile razor blade. The resulting minced tissue was then enzymatically digested for 60 min at 37 °C with 0.15% (*w/v*) collagenase type I. At the completion of the incubation, alpha-minimal essential medium (α-MEM) containing 10% (*v/v*) FBS was added to the resulting digested solution, which was then filtrated through a 70-μm filter in order to discard undissociated tissue. The filtrate was then centrifuged at 800× *g* for 5 min. The pellet was then resuspended in α-MEM medium containing 10% (*v/v*) FBS and 1% (*v/v*) antibiotics solution. The resulting culture was then incubated in standard conditions for cell culture and passaged with trypsin/EDTA when confluent [12,18].

#### 2.2.2. uAD-SC Differentiation into a Schwann Cell Phenotype (dAD-MSC)

After uAD-MSCs reached 60% confluency, the α-MEM medium containing 10% (*v/v*) FBS and 1% (*v/v*) antibiotics solution was removed and replaced with fresh medium containing 1 mM β-mercaptoethanol and incubated in standard conditions for cell culture for 24 h. The cells were then washed with Hanks’ buffered salt solution (HBSS) and further incubated for 72 h in the α- MEM growth medium containing 35 ng/mL all-trans-retinoic acid. The cells were then washed and the medium was replaced with α- MEM growth medium supplemented with 5 ng/mL platelet-derived growth factor (PDGF), 10 ng/mL basic fibroblast growth factor (bFGF), 14 μM FSK, and 126 ng/mL GGF-2 (glial differentiation medium). The uAD-MSCs were cultured for 2 weeks in the glial differentiation medium, after which they were stained for glial markers: glial fibrillar acidic protein (GFAP), p75 neurotrophin receptor (p75NTR), and S100 (see Supplementary Materials, Figure S1) [49,51].

### 2.3. Flow Cytometry

When cells were approximately 70–80% confluent, the flasks were washed with HBSS and incubated 5 min at 37 °C in Accutase solution® (Sigma-Aldrich) for cell detachment. Following centrifugation at 900 rpm for 5 min, cell pellets were washed with 10 mM phosphate buffer saline (PBS). For CD44 staining, cells were processed alive, whereas for RHAMM staining, cell pellets where resuspended in 4% paraformaldehyde (PFA) for 20 min at room temperature as suggested from the antibody manufacturer. The cells were resuspended in blocking buffer (10% FBS in PBS for CD44 or 10% of normal goat serum NGS, for RHAMM staining) and incubated for 10 min at RT. Cells were further centrifuged and pellet were washed with flow cytometry buffer (1% FBS in PBS). For CD44 staining, cells were incubated for 30 min on ice with a FITC conjugated mouse monoclonal anti-Rat CD44 antibody (1:50, BD Biosciences) and washed twice in flow cytometry buffer before analysis with Cyan ADP flow cytometer (Beckman Coulter, High Wycombe, UK). During the analysis, the dead cells were gated out by propidium iodide staining (1 mg/mL). For RHAMM staining, PFA-fixed cells were incubated for 30 min on ice in mouse monoclonal anti-RHAMM (1:50, Abcam, Cambridge, UK), washed in flow cytometry buffer, and incubated 30 min on ice in goat anti-mouse Alexa 488® conjugated secondary antibody (1:500, Life Technologies, Warrington, UK), before proceeding with flow cytometry analysis. Data were analyzed with FlowJo vX.0.7 (Tree Star Inc., San Carlos, CA, USA). Median fluorescence intensity (MFI) fold change was calculated by normalizing for the isotype/no primary controls for each cell type.

### 2.4. Scratch Wound Assay (SWA)

Primary nSCs, aSCs, uAD-MSCs, and dAD-MSCs were harvested as previously described and seeded on 5 cm TCPS Petri dishes (pre-coated with poly(D-lysine) for SC cultures), in full medium specific to cell type and incubated in standard conditions for cell culture. When the cells reached 100% confluency, the medium was removed and replaced with plain medium with or without blocking agents (anti-RHAMM and/or anti-CD44 antibody at 30 μg/mL) and incubated for 30 min in standard conditions for cell culture. The medium was then removed and washed with PBS three times, for 5 min at RT, and a scratch (600 to 800 μm) was done in the middle section of the cell culture. Medium with or without HA [MW: 234 kDa, 0.2 mg/mL] was then added to the cell culture, and cell motility from the edge to the center of the scratch was monitored for 24 h with an AS MDW live cell imaging system (Leica) using a (63x/1.30 Plan Apo glycerine) objective, the (BGR) filter set (Chroma (61002)), and a (red (DS Red)) Precise LED fluorescent light source. Imaging software Image Pro 6.3 by Media Cybernetics LtD (Rockville, MD, USA). Point visiting was used to allow multiple positions to be imaged within the same timecourse and cells were maintained at 37 °C and 5% CO_2_. The images were collected using a (Coolsnap HQ (Photometrics)) camera with a Z optical spacing of (0.2 μm) and only the (maximum intensity) projections of these images are shown in the results. The cell velocity was assessed using the TScratch program (Tobias Gebäck and Martin Schulz, ETH Zürich; https://www.cse-lab.ethz.ch/software/).

Samples were fixed and stained for RHAMM and CD44 as previously described, except for the primary antibody and secondary antibody used to stain CD44 (in this experiment, primary antibody: OX50 clone monoclonal anti-CD44 antibody, Alexa 568 goat anti-mouse antibody), and α-tubulin 1 h and 24 h after the scratch in order to investigate HA receptors expression and tubulin polymerization of the cells on the edge of the scratch. For the tubulin staining, the cells were fixed as previously described after 1 and 24 h incubation after the wound was created, pre-incubated in medium containing HA or not, and pre-treated or not with anti-RHAMM and/or anti-CD44 as previously described. The cells were then washed and blocked with 5% goat serum in PBS for 1 h at RT, washed further, and permeabilized with 0.5% saponin in PBS for 30 min at RT. The cells were then incubated with primary polyclonal rabbit anti-α tubulin, washed three times with PBS, and incubated with goat anti-rabbit Alexa Fluor 350, RT, in the dark. The cells were washed again three times for 5 min with PBS, and mounted with anti-fade reagent. Pictures of the samples were then processed using same live-imaging microscope as previously described.

### 2.5. Statistical Analysis

Statistical significance of the results of flow cytometry analysis was evaluated by one-way analysis of variance (ANOVA) with Tukey’s multiple comparison tests using GraphPad Prism 6 (GraphPad Software Inc., San Diego, CA, USA). Levels of significance were expressed as *P*-values (* *P* < 0.05, ** *P* < 0.01).

## 3. Results and Discussion

### 3.1. HA and HA Receptors Are Abundant in Peripheral Nerves

**Figure 1:**
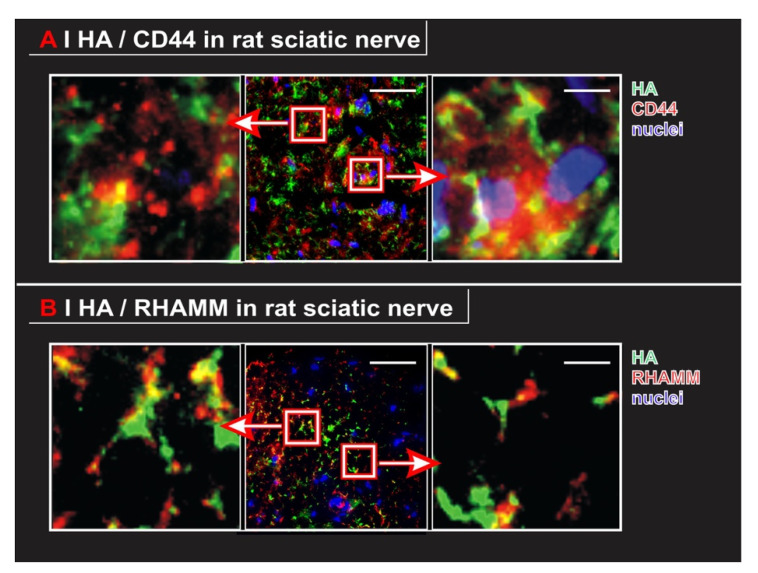
Presence of hyaluronic acid (HA) and its receptors in sciatic nerves. (**A**) HA and CD44 localization in cross-sections of sciatic nerves of adult male SD rats by fluorescence histocytochemistry. HA was imaged using a biotinlylated hyaluronic acid binding protein (HABP) and then FITC-labelled streptavidin (green), CD44 with mouse monoclonal anti-CD44 (red), cell nuclei with DAPI (blue). The scale bar in the central image corresponds to 30 μm, in the insets to 5 μm. (**B**) Distribution of HA and RHAMM as above. RHAMM was imaged using a mouse monoclonal anti-CD44 (red).

The currently available information about the role of HA and its receptors in the PNS are rather scarce. CD44 has been proposed as a glial marker e.g., for retina glial cells (Müller cells) [52], non-myelinating Schwann cells [53], and differentiating astrocytes [54,55]. In the sciatic nerve of rats, HA presence has been reported in myelin sheaths [56], and CD44 (higher in neonatal than in adult rats) seems to be involved in Schwann cell-neurite adhesion [57] and to be expressed in larger amounts in Schwann cells upon injury [38,58]. We can indeed confirm that HA is abundant in the sciatic nerves of adult male SD rats (Figure 1; see also histochemical analysis in Supplementary Materials, Figures S2 and S3). Further, both CD44 and RHAMM are clearly present, although their association/colocalization with HA is moderate; to our knowledge, this is the first observation of RHAMM in peripheral, non-tumor-bearing nerves. Please note that CD44 is also visible far from cells, but this is not surprising because it can be present in soluble forms.

### 3.2. RHAMM but Not CD44 Is Upregulated in the Differentiation of Progenitor Cells (uAD-MSCs) to a Schwann-Like Phenotype (dAD-MSCs)

**Figure 2:**
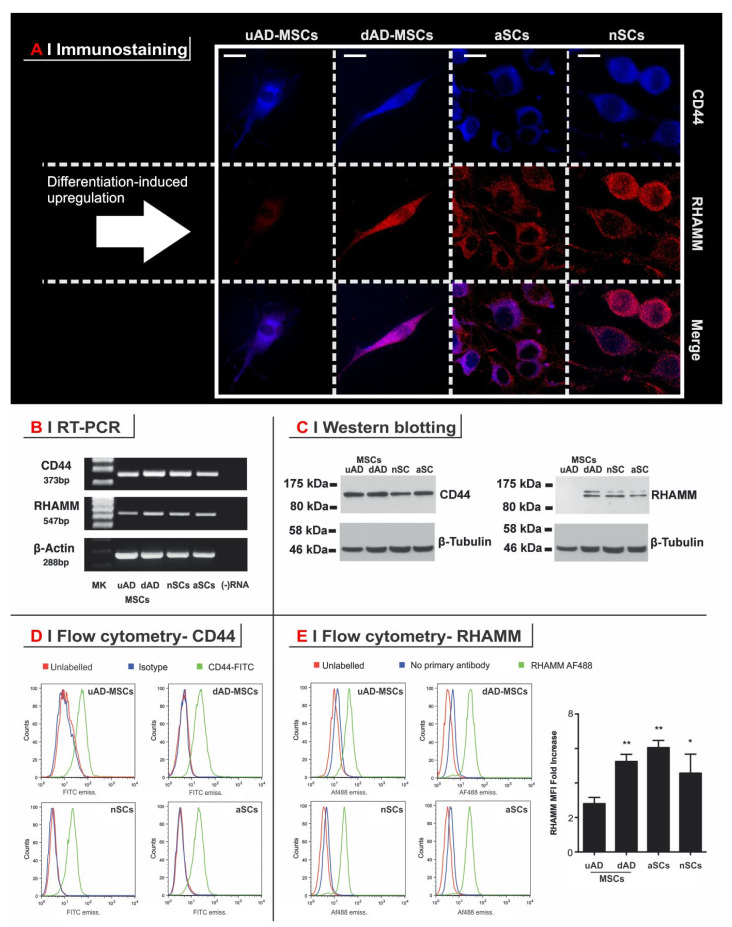
Expression of HA receptors in Schwann and Schwann-like phenotypes. (**A**) Immunostaining of CD44 and RHAMM in non-permeabilized nSC (primary neonatal Schwann cells), aSC (primary adult Schwann cells), uAD-MSCs (undifferentiated adipose stem cells), dAD-MSCs (differentiated adipose stem cell into a Schwann cell phenotype) (×60). Blue: CD44, red: RHAMM. The scale bars correspond to 25 μm; please note that the location of the two receptors is maximal in the central region of the cell bodies, which appears to suggest a round shape of the cells; on the contrary, these cells are much elongated (see also the controls in Figure 3B or the movie uploaded to exemplify the scratch wound assay. (**B**) Gene expression of CD44 (top) and RHAMM (middle) via semi-quantitative RT-PCR in the same cell types. Specific primers for β-actin were used to ensure equal RNA loading. (**C**) Western blot analysis of CD44 (left) and RHAMM (right); for RHAMM, we here report only the most constant band corresponding to a molecular weight ≥90 kDa. (**D**) Flow cytometry analysis of CD44; no significant difference can be recorded among the cell types in relation to its expression. (**E**) Flow cytometry analysis of RHAMM; the median fluorescence intensity (MFI, normalized to control without primary antibody) fold change increase for uAD-MSCs was significantly lower (2.800 ± 0.208) than those of dAD-MSCs (5.247 ± 0.237, ** *p* < 0.01), aSCs (6.050 ± 0.236, ** *p* < 0.01), and nSCs (4.567 ± 0.636, ** *p* < 0.01). (*n* = 3). The difference was considered statistically significant when *p*-value < 0.05 (* *p* < 0.05).

Since CD44 expression is widely reported both in AD-MSCs [59,60,61] and in Schwann cells [38,57,58], it is reasonable to expect its levels not to be much affected by the differentiation of the former to a Schwann-like phenotype. Indeed, immunostaining, RT-PCR, Western blotting, and flow cytometry (Figure 2) showed no significant difference in CD44 presence between rat undifferentiated AD-MSCs (uAD-MSCs), Schwann-like cells AD-MSCs (dAD-MSCs), neonatal Schwann cells (nSCs), and adult Schwann cells (aSCs).

Regarding RHAMM, literature offers hardly any data about its expression in PNS glia; our analysis indicates an important difference between uAD-MSCs and all other cell types. Firstly, in uAD-MSCs, RHAMM was not detected via immunostaining (Figure 2A) and Western blotting (Figure 2C), which on the contrary, showed high and comparable levels of this protein through the other cell types. Secondly, flow cytometry (Figure 2D) showed a roughly 2-fold increase in RHAMM between uAD-MSCs and dAD-MSCs, which on their turn again were similar to Schwann cells (Figure 2E). Last, RHAMM transcripts (547 bp) were found in all cell types, but its gene expression levels appeared lower in uAD-MSCs than in the other cell types (Figure 2B).

Therefore, despite some differences among the analytical techniques, RHAMM was shown to be significantly upregulated upon uAD-MSC differentiation to dAD-MSCs; the latter cells not only express typical glial markers (see Supplementary Materials, Figure S1) but also show RHAMM levels indistinguishable from those of Schwann cells (no difference recorded between nSCs and aSCs).

It is worth pointing out that the number and identity of RHAMM isoforms is variable, and several of them have been recorded in glial cells. For example, in culture, it has been shown that astrocytes produce a 72 kDa isoform, while microglia at least three with molecular weights of 50, 65, and 80 kDa (50 kDa being the major one) [62]. In addition, 75 and 90 kDa isoforms have been identified in rat brain homogenates, followed by a third one at 66 kDa [63,64].

This study does not focus on the specific identification of the RHAMM isoforms, but it must be noted that RHAMM always presented a double band at ≥90 kDa in dAD-MSCs, nSCs, and aSCs. Often two other isoforms could be detected at about 50 and 70 kDa (the latter almost exclusively seen in aSCs, and all being absent in uAD-MSCs), but in a more variable fashion. Preliminary experiments may indicate a predominant cytoskeletal localization for the 50 kDa form, and on membranes for the other forms (see Supplementary Materials, Figures S4 and S5).

### 3.3. HA Receptors Affect Independently Both Motility and Morphology of Schwann (-Like) Cells

**Figure 3:**
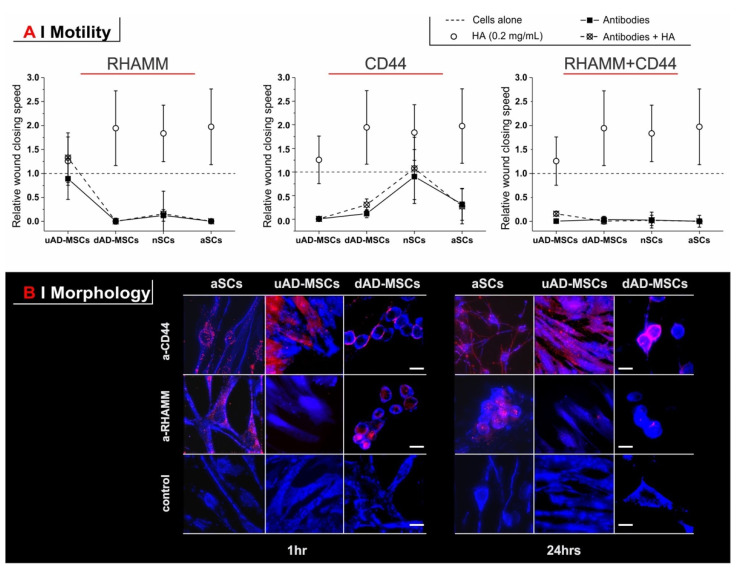
Effects of blocking HA receptors on motility and morphology. (**A**) Relative wound closure rates for aSCs, nSCs, uAD-MSCs, and dAD-MSCs; the actual would closure rates (see those for neonatal and adult SCs as examples in Supplementary Materials, Figure S4) were obtained as the % decrease of the wound area per time unit through the use of the Tscratch software (see Experimental Section, panel B), using images recorded every 10 min through 24 h. The relative wound closing rates were obtained by normalizing the actual rates by dividing them by the closing rate of the control. Please note that in the controls, the would closing rate of uAD-MSCs and dAD-MSCs is indistinguishable (around 1.6% wound area/h) and slightly lower than that of aSCs and nSCs (resp. 2.3 and 2.6% wound area/hour) although the difference is not statistically significant. (*n* = 3) (**B**) Immunostaining of SCs, uAD-MSCs, and dAD-MSCs located at the edge of the scratch, 1 and 24 h after the scratch (×60). The cells were pre-treated with anti-RHAMM or anti-CD44 (both in red), and co-stained for tubulin (blue). The scale bars correspond to 25 μm.

The key role played by HA receptors in cell motility is well known, although literature does not always report unequivocal effects; for example, blocking RHAMM decreases the migration of smooth muscle cells (after injury) [65] and of endothelial cells (in in vitro angiogenesis) [66], but in the latter case, blocking CD44 seems to have no effect. In other cases, CD44 blockage has been clearly shown to impair cell motility (e.g., in melanoma [67]). In the CNS, glial cell migration is reportedly impaired by blocking either RHAMM [62] or CD44 [37]. In short, depending on the cell type, motility may depend more on one or the other receptor, or equally on both. Here, we have investigated the motility of nSCs, aSCs, dAD-MSCs, and uAD-MSCs by the means of a scratch wound assay (Figure 3A; Supplementary Materials Video S1 showing the assay on aSCs with and without HA or the different antibodies), with the following observations:

(a) The supplementation of the cell culture medium with HA (234 kDa, 0.2 mg/mL; empty circle in all panels of Figure 3A) doubled the scratch closure rate for dAD-MSCs, nSCs, and aSCs, i.e., all the cell RHAMM(+) types. uAD-MSCs presented a modest (about 30%) but not statistically significant increase.
(b) Anti-RHAMM almost completely immobilized the three RHAMM(+) types, but not uAD-MSCs, whose motility was unaffected by this blocking antibody (Figure 3A, left panel). Interestingly, supplementation of HA + anti-RHAMM had on uAD-MSCs the same modest stimulatory effect seen for HA alone, whereas had no effect on the other cell types. These data are in agreement with the substantial absence of RHAMM in uAD-MSCs and support the hypothesis of RHAMM as a gatekeeper for HA-mediated motility effects.
(c) The effect of anti-CD44 was largely different; this blocking antibody abrogated the motility of uAD-MSC, appreciably reduced that of dAD-MSCs and aSCs (20–30% of initial motility) and had marginal influence on nSCs, but with largely variable results. Since the overall levels of CD44 (all standard isoform) did not appear to be much different among the cell types, the scarce sensitivity of nSCs to anti-CD44 may be due to a specific post-translational modification of this receptor. In any case, the key observation is that also CD44 blockage reduced motility, but through mechanisms quite different from RHAMM blockage. It is noteworthy that the RHAMM(+) cell types had some gain in wound closing speed in the presence of HA, also in the presence of anti-CD44, although the effect was marginal in comparison to the non-blocked controls.
(d) Anti-CD44 and anti-RHAMM together had an additive (if not synergic) effect; all cell types were immobilized by their combination, independent of the presence of HA.

Motility necessarily requires cells to adhere to a substrate; it can be argued that the antibodies may affect the first by reducing the second, which may cause cells to adopt a rounder phenotype. We investigated morphological effects through a tubulin stain, while at the same time, highlighting the antibody binding by using fluorescently labelled anti-RHAMM and anti-CD44 (Figure 3B):

(a) Anti-RHAMM did not appreciably bind to uAD-MSCs, thereby confirming the RHAMM(low) nature of these cells and supporting the lack of effects of the antibody treatment. Correspondingly, the cells preserved an elongated/stretched morphology.
(b) Curiously, anti-CD44 clearly bound (and immobilized) uAD-MSCs did not appear to cause any significant morphological alteration to them. This would appear to indicate that the anti-CD44 effect on these cells may be due to specific signaling rather than to a decreased adhesion to substrate.
(c) The RHAMM(high) cell types showed similar effects upon anti-RHAMM and anti-CD44 treatment: the elongated/stretched phenotype was lost and the cells acquired a more round morphology. It is noteworthy, however, that the two antibodies had virtually identical effects on the motility of the two cells, but dAD-MSCs were round already after 1 h of exposure, while aSCs required a longer time (effect apparent at 24 h); due to the different kinetics of the reduction in adhesion, also in this case, we are tempted to ascribe the effects on motility to a signaling cascade triggered by the receptor binding rather than to loss of adhesion.

The above observations therefore confirm the RHAMM-dependency of both motility and morphology of dAD-MSCs, similarly to SCs and differently from uAD-MSCs. Our data also suggest that RHAMM and CD44 use complementary circuits to affect motility, with RHAMM possibly more critical than CD44 for Schwann(-like) cells. Last, the role of both receptors on motility may not be directly linked to them being involved in the adhesion to the substrate, although it must be emphasized that this result strictly refers to poly(D-lysine)-coated plastics.

## 4. Conclusions

We have demonstrated that RHAMM expression is high in Schwann cells and it is also a peculiarity of the Schwann-like differentiation of AD-MSCs. Further, we have shown that RHAMM is critical for the motility of these RHAMM(high) cells, whereas CD44 is apparently more critical for that of the RHAMM(low) uAD-MSCs; while it is clear that both HA receptors have key roles in cell motility, our data appear to support literature data (not on PNS cells) that their stimulation is complementary and has cell-dependent effects. The results of this study offer some important indications in the context of a peripheral nerve regenerative therapy: Firstly, that the motility also of freshly differentiated Schwann-like cells can be boosted by some form of supplementation with HA; secondly, that attention must be paid to develop tools (e.g., artificial extracellular matrices based on HA) that can maintain a correct RHAMM(high) phenotype and a continuous stimulation of this receptor.

## Supplementary Materials

The following are available online at https://www.mdpi.com/2073-4409/9/6/1477/s1, Figure S1: Glial markers expression, Figure S2: Distribution of HA, CD44 and RHAMM in cross-sections of sciatic nerve from adult male SD rats by fluorescence histocytochemistry, Figure S3: Distribution of HA in cross-sections of sciatic nerve from adult male SD rats by fluorescence histocytochemistry, Figure S4: Western blotting analysis of RHAMM on subcellular fractions of aSCs (top) and nSCs (bottom); Figure S5: Controls for the intracellular fractionation experiments, Figure S6: Wound closure rate for neonatal (left) and adult Schwann cells (right) as a function of the presence of HA or of antibodies for RHAMM or CD44, Table S1: Sequences of primers for the RT-PCR studies, Additional experimental information is provided as supplementary information., Video S1: An mp4 video of scratch wound assays conducted on ASCs (control, HA, anti-CD44 and anti-RHAMM) is provided as additional material, Methods S1: Materials, Methods S2: Histochemistry, Methods S3: Reverse- Transcriptase PCR (RT-PCR), Methods S4: Immunocytochemistry, Methods S5: Western blotting.
