# Supplementary material for: Hyaluronic Acid (HA) Receptors and the Motility of Schwann Cell(-Like) Phenotypes
Source: Cells. 2020 Jun 17;9(6):1477. doi: 10.3390/cells9061477 (PMC7349078; doi:10.3390/cells9061477)
Supplement: Supplementary file 1 [file cells-09-01477-s001.zip › cells-801127-supplementary/Supp_info_Schwan[2].docx]

# **Hyaluronic acid (HA) receptors and the motility of Schwann cell(-like) phenotypes**

## Supplementary Materials

Sihem Ouasti^1,§†^, Alessandro Faroni^2†^, Paul J. Kingham^2,3^, Matilde Ghibaudi^4^, Adam J. Reid^2,5^, Nicola Tirelli^1,4^

^1^ Division of Pharmacy and Optometry, School of Health Sciences, Faculty of Biology Medicine and Health, University of Manchester, Manchester Academic Health Science Centre, Manchester M13 9PL, UK.

^2^  Blond McIndoe Laboratories, Division of Cell Matrix Biology and Regenerative Medicine, School of Biological Sciences, Faculty of Biology Medicine and Health, University of Manchester, Manchester Academic Health Science Centre, Manchester M13 9PL, UK.

^3^ Department of Integrative Medical Biology, Section of Anatomy, Umeå University, 901 87 Umeå, Sweden.

^4^ Laboratory of Polymers and Biomaterials, Fondazione Istituto Italiano di Tecnologia, 16163 Genova, Italy

^5^ Department of Plastic Surgery & Burns, Wythenshawe Hospital, Manchester University NHS Foundation Trust, Manchester Academic Health Science Centre, Manchester M23 9LT, UK.

^†^ The two authors contributed equally

**Materials and methods**

**S1 Materials**

Sprague-Dawley male rats (SD male rats) and pregnant female rats (SD pregnant female rats) were bought from Harlan Laboratories (UK). All the experiments involving animals were carried out in accordance with the U.K. Animals (Scientific Procedures) Act, 1986. Tissues were harvested from the animals following terminal anesthesia with CO_2_ and cervical dislocation. Dulbecco’s modified eagle medium (DMEM), antimycotic and antibiotics reagent, forskolin, poly (D-lysine) were bought from Sigma-Aldrich (Poole, Dorset, UK). Alpha Modified Eagle Medium (α-MEM), foetal bovine serum, glutamate, trypsin-EDTA, Hanks balanced salt solution (HBSS) and collagenase type I were purchased from Life Technologies (Paisley, UK). Phosphate buffer saline was bought in pellets from Oxoid (Basingstoke, UK).

Goat serum, saponin, β-mercaptoethanol, picric acid, all-trans-retinoic acid and sodium azide were purchased from Sigma-Aldrich (Poole, Dorset, UK). Secondary antibodies Alexa Fluor 350 goat anti-rabbit and Alexa Fluor 568 goat anti-mouse were purchased from Life Technologies (Paisley, UK). RNeasy kit, DNase I, RNase free water, one-step RT-PCR kit were purchased from Qiagen (Crawley, UK). Paraformaldehyde and sucrose were bought from VWR (Lutterworth, UK). Rabbit polyclonal sodium potassium ATPase antibody, mouse monoclonal vimentin antibody, rabbit polyclonal HDAC1 antibody, rabbit polyclonal HP1-α antibody and polyclonal rabbit anti-CD44 antibody were purchased from Abcam (Cambridge, UK). Avidin/biotin blocking solutions, biotinylated hyaluronic acid binding protein (HABP), fluorescein-conjugated streptavidin, secondary antibody goat anti-rabbit FITC and horse anti-mouse FITC, Vectashield Mounting Medium with DAPI were purchased from Vector Laboratories (Peterborough, UK). Platelet derived growth factor (PDGF) and basic fibroblast growth factor (bFGF) were bought from Sera Laboratories Int. (Haywards Heath, UK). Mouse monoclonal HSP90 antibody, unlabelled and non-specific IgG and rabbit polyclonal RHAMM antibody were bought from Santa Cruz Biotechnology (Heidelberg, Germany). Mouse monoclonal RHAMM antibody was bought from Leica Microsystems (Milton Keynes, UK). OX-50 monoclonal antibody anti-CD44 was purchased from Millipore (Billerica, MA). Glial growth factor (GGF-2) was a kind gift from Acorda Therapeutics Inc. (Hawthorne, NY.). HRP-conjugated secondary antibodies goat anti-mouse and goat anti-rabbit (Cell Signalling Technology, Danvers, MA). OCT (optimal cutting temperature) medium was purchased from Cellpath (Newtown, UK). The DC protein assay was purchased from Biorad (Hemel Hempstead, UK). S100 rabbit polyclonal antibody was purchased from Dako (Carpinteria, CA, USA). P75NTR rabbit polyclonal antibody was purchased from Promega (Madison, WI, USA).

The Subcellular Protein Fractionation kit and GFAP mouse monoclonal antibody were purchased from ThermoFisher scientific (Nortumberland, UK).

**S2 Histochemistry**

Sciatic nerves were harvested from SD male rats and fixed overnight at 4°C in Zamboni fixative (85 mL 2 % paraformaldehye in PBS, 15 mL saturated picric acid). After fixation, the tissue was transferred in 0.01 M PBS containing 15 % (w/v) sucrose and 0.1 % (w/v) sodium azide, which was changed several times over the period of 1 to 2 days, until the solution was clear and the tissue had sunk to the bottom of the container. The sciatic nerve was then embedded in OCT (optimal cutting temperature medium for cryosections) and frozen in liquid nitrogen. Following freezing of the samples, the cross-sections of a thickness of 15 μm were produced in a cryostat.

In order to fluorescently label endogenous hyaluronic acid (HA) in the sciatic nerve, the cross-sections were placed in a wet box and treated successively with an avidin and a biotin solution for 20 min at room temperature (RT) in order to block any endogenous avidin/biotin activity. After washing with PBS, the samples were incubated in 0.1 % (w/v) bovine serum albumin in PBS for 1 hour at RT. The samples were then washed in PBS and incubated further with a solution of biotinylated hyaluronic acid binding protein in PBS (2 μg/mL) for 1 to 2 hrs, at RT. At the completion of the incubation, the samples were washed three times for 10 min with PBS and further incubated with fluorescein-conjugated streptavidin for 15 min at RT. The samples were then rinsed in PBS for 5 min and incubated with 5 % goat serum in PBS for 1 hour at RT, washed again and incubated with mouse monoclonal anti-CD44 (20 μg/mL) for 1 hour at RT, or with mouse monoclonal anti-RHAMM (20 μg/mL) for 1 hour at RT and washed four times for 10 min with PBS. The samples were then incubated with goat secondary anti-mouse antibody Alexa Fluor 568 (8 μg/mL) in PBS for 1 hour at RT and washed four times for 10 min with PBS. The cross-sections were finally mounted in Vectashield anti-fade mounting gel containing DAPI. The samples were then visualised on a Delta Vision RT (Applied Precision) restoration microscope using a [*60x/ 1.42 Plan Apo*] objective and the [*Sedat*] filter set (Chroma [*89000*]). The images were collected using a Coolsnap HQ (Photometrics) camera with a Z optical spacing of 0.2 μm. Raw images were then deconvolved using the Softworx software; [*maximum intensity*] projections of the deconvolved images are shown in the results.

**S3 Reverse-****Transcriptase PCR (RT-PCR)**

For each preparation, cells were harvested from confluent cultures on TCPS by treatment with trypsin/EDTA. The cells were homogenized, and total RNA was extracted using *RNeasy Mini Kit* (Qiagen) following manufacturer’s instructions. After treatment of the samples, with *DNAse I* (Qiagen) and dissolving the samples in *RNAse free water* (Qiagen), the RNA concentration of the different cell batches was measured by its UV absorption at 260-80 nm, and RT-PCR was performed using the *One-step RT-PCR Kit* (Qiagen). The RHAMM primer chosen corresponded to sequences conserved in murine and human RHAMM.[1-3] The primers were manufactured by Sigma, UK (Table 1). A thermocycler (MJ Research PTC-200 Thermo Cycler, GMI Ltd., MN, USA.) was used with the following parameters: a reverse transcription step (50°C, 30 min), a nucleic acid denaturation/reverse transcriptase inactivation step (95°C, 15 min) followed by 35 cycles of denaturation (95 °C, 30 s), annealing (60 °C, 30 s) and primer extension (72°C, 1 min) followed by final extension incubation (72 °C, 5 min). PCR amplicons were separated via electrophoresis (50 V, 90 min) through a 1.5% (w/v) agarose gel and the size of the PCR products was estimated by using Hyperladder IV (Bioline, UK). Samples were visualized under UV illumination following GelRed nucleic acid stain (BioNuclear, Sweden) incorporation into the agarose.

**S4. Immunocytochemistry**

Subconfluent cultures of nSCs, aSCs, uAD-MSCs and dAD-MSCs were trypsinized and seeded in slide-flasks (Thermofisher) at a density of 10,000 cells/cm^2^, and left overnight to incubate in standard conditions for cell culture in their respective (as described there above) culture medium. The medium was then removed from the slide-flask and the cells were washed with ice-cold PBS, fixed with 4 % paraformaldehyde (w/v) in PBS for 30 min at RT, washed with PBS and incubated for 1 hour with 5 % goat serum in PBS in order to block any immuno-cross reactions, and washed further with ice-cold PBS. The cells were then incubated with monoclonal anti-RHAMM antibody (20 μg/mL) for 1 hour, RT, in the dark, washed three times with PBS for 10 min and further incubated with secondary antibody Alexa 568 goat anti-mouse antibody. Cells were then washed a further three times with PBS and incubated with primary rabbit polyclonal anti-CD44 (2 µg/mL) for 1 hour, RT, in the dark and washed three times with PBS for 10 min followed by incubation with secondary antibody Alexa 350 goat anti-rabbit antibody. Cells were finally washed further with PBS three times for 10 min and the slides were mounted using an anti-fade mounting reagent. Images were acquired on a Delta Vision RT (Applied Precision) restoration microscope using a [*60x/ 1.42 Plan Apo*] objective and the [*Sedat*] filter set (Chroma [*89000*]). The images were collected using a Coolsnap HQ (Photometrics) camera with a Z optical spacing of 0.2μm. Raw images were then deconvolved using the Softworx software and [*maximum intensity*] projections of these deconvolved images are shown in the results.

**S5. Western blotting**

For each preparation the cells were cultured on TCPS until reaching 80-90 % confluency. The cells were then washed with ice-cold PBS, scrapped and pelleted in lysis buffer containing 20mM 4-(2-hydroxyethyl)- 1-piperazineethanesulfonic acid (HEPES, pH 7.4) buffer, 100 mM NaCl, 1% (v/v) Nonidet P-40, 5 mM EDTA and a cocktail of protease and phosphatase inhibitors (Thermo Scientific, Loughborough, UK). The protein concentration was determined using the DC Protein Assay (Biorad). Samples were then diluted in lysis buffer and 2x SDS reducing buffer (Tris-HCl 100 mM, pH 6.8, SDS 4% [w/v], bromophenol blue 0.2% [w/v], glycerol 20% [v/v], b-mercaptoethanol 200 mM) and finally heated to 95°C for 5 min. Equal amounts of protein (30 μg/well) were loaded onto 10% (w/v) SDS-polyacrylamide gels and separated via electrophoresis at 200 V for 1 hour. The separated proteins were transferred to Hybond ECLTM nitrocellulose membranes (GE Healthcare, Buckinghamshire, UK) at 80 V for 1 hour. The membranes were gently agitated for 1 hour in blocking buffer containing 5% (w/v) nonfat milk powder in blotting buffer (10 mM Tris-base, 100 mM NaCl, 0.1% Tween, pH 7.5) and were further incubated overnight at 4°C with primary antibody (anti-CD44 mouse monoclonal, 1:500; anti-RHAMM rabbit polyclonal, 1:500). At the completion of incubation, the membranes were washed six times for 5 min, in blotting buffer and incubated further for 1 hour with horseradish peroxidase-(HRP) conjugated secondary antibody anti-mouse (1:1000, to detect CD44) or anti-rabbit (1:1000, to detect RHAMM) for 90 min, and washed again as previously described. The membranes were then treated with SuperSignal West Pico Chemiluminescent Substrate (Thermo Scientific, UK) for 5 minutes and exposed to Amersham Hyperfilm for signal detection (GE Healthcare, Buckinghamshire, UK). The blots were then stripped for 30 minutes at RT with a stripping buffer (Thermo Scientific, UK), re-blocked with 5 % non-fat dry milk and incubated overnight at 4ºC with a rabbit polyclonal anti β-tubulin antibody to ensure that samples have been equally loaded. The following day membranes were washed, incubated with a HRP-conjugated anti-rabbit secondary antibody (1:2000), and treated with SuperSignal West Pico Chemiluminescent Substrate for chemiluminescence detection on Amersham Hyperfilm.


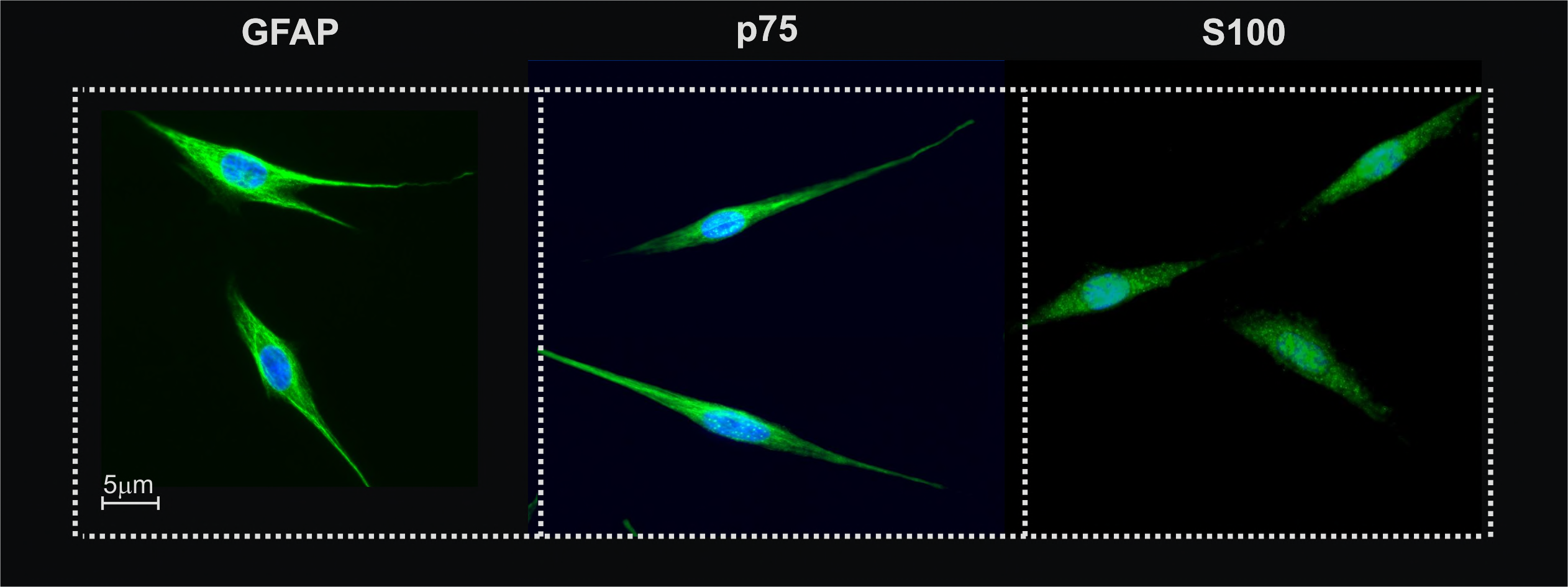


**Figure S1:** Glial markers expression. After 2 weeks of culture in a differentiation medium containing glial growth factors, dADSC were found to express GFAP, p75 and S100. Green: GFAP, p75NTR, and S100. Blue: DAPI**.**


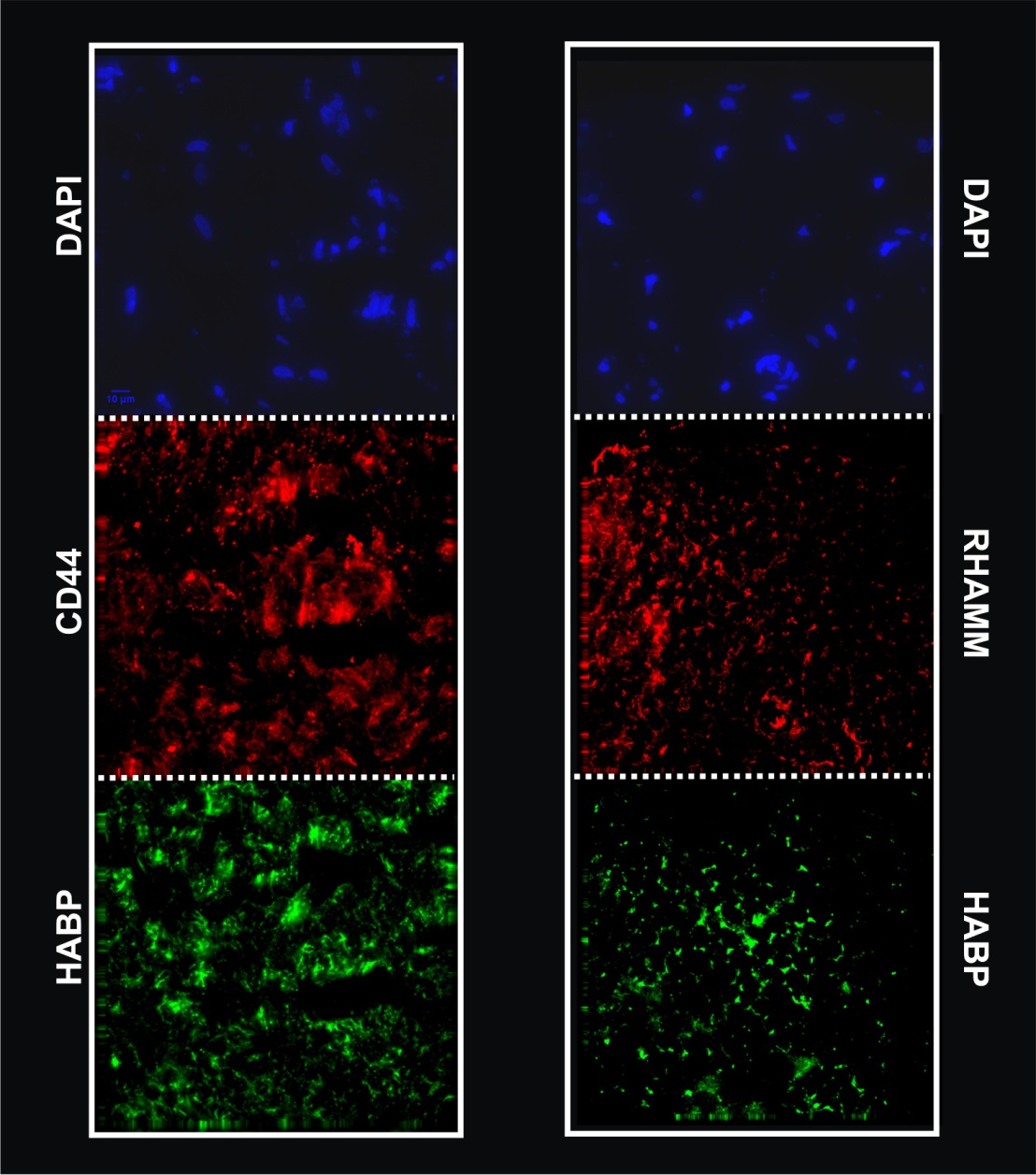


**Figure S2.** Distribution of HA, CD44 and RHAMM in cross-sections of sciatic nerve from adult male SD rats by fluorescence histocytochemistry. *Left:* *DAPI* (blue): localization of cell nuclei. *HABP* (green): localization of HA (fluorescein-labelled streptavidin bound to biotinylated hyaluronic acid binding protein, HABP). *CD44* (red): localization of CD44 (Alexa Fluor 568-labelleld goat secondary anti-mouse antibody bound to mouse monoclonal anti-CD44). *Right:* as for the left column, replacing CD44 with RHAMM.


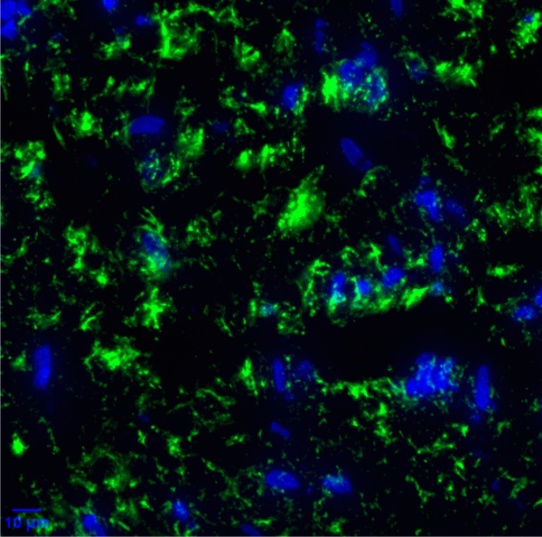


**Figure S3.** Distribution of HA in cross-sections of sciatic nerve from adult male SD rats by fluorescence histocytochemistry. *DAPI* (blue): localization of cell nuclei. *HABP* (green): localization of HA (fluorescein-labeled streptavidin bound to biotinlylated hyaluronic acid binding protein, HABP).


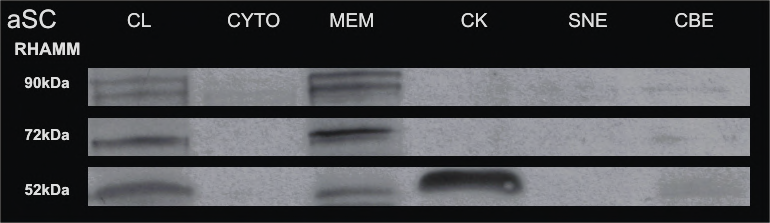


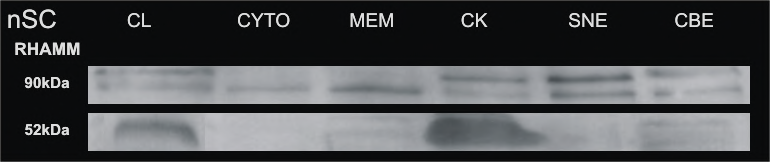


**Figure S4.** Western blotting analysis of RHAMM on subcellular fractions of aSCs (top) and nSCs (bottom). CL: cell lysate; CYTO: cytoplasmic fraction; MEM: membrane fractions (plasma membrane, endoplasmic reticulum and Golgi); CK: cytoskeleton fraction; SNE: soluble nuclear extract; CBE: chromatin-bound extract.


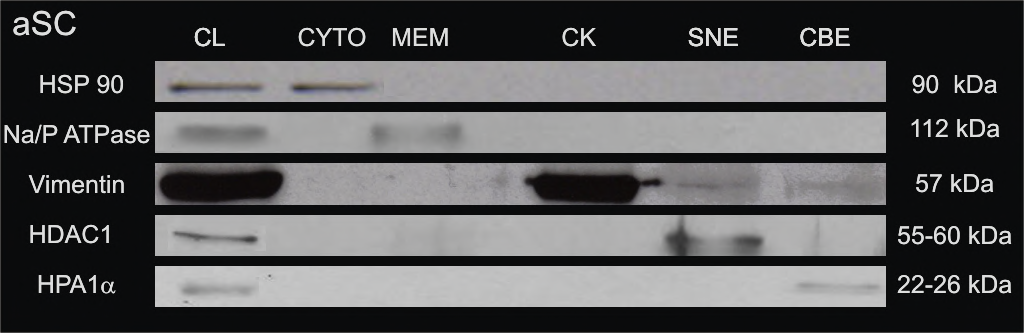


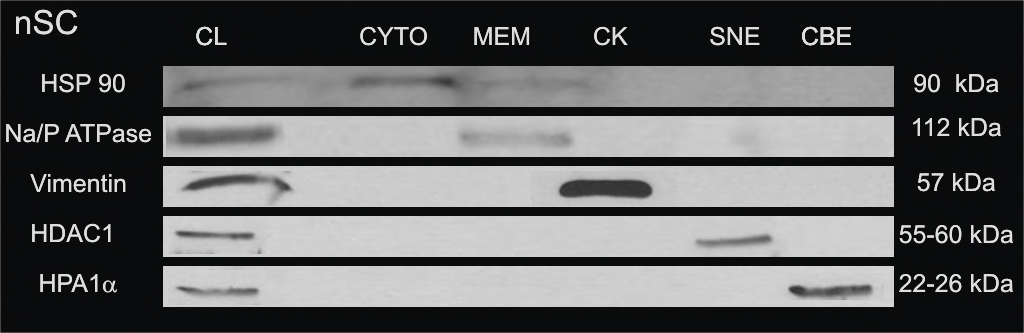


**Figure S5.** Controls for the intracellular fractionation experiments. MEM: membrane fractions (plasmic membrane, reticulum endoplasmic and golgi), CK: cytoskeleton fraction, SNE: soluble nuclear extract, CBE: chromatin-bound extract.


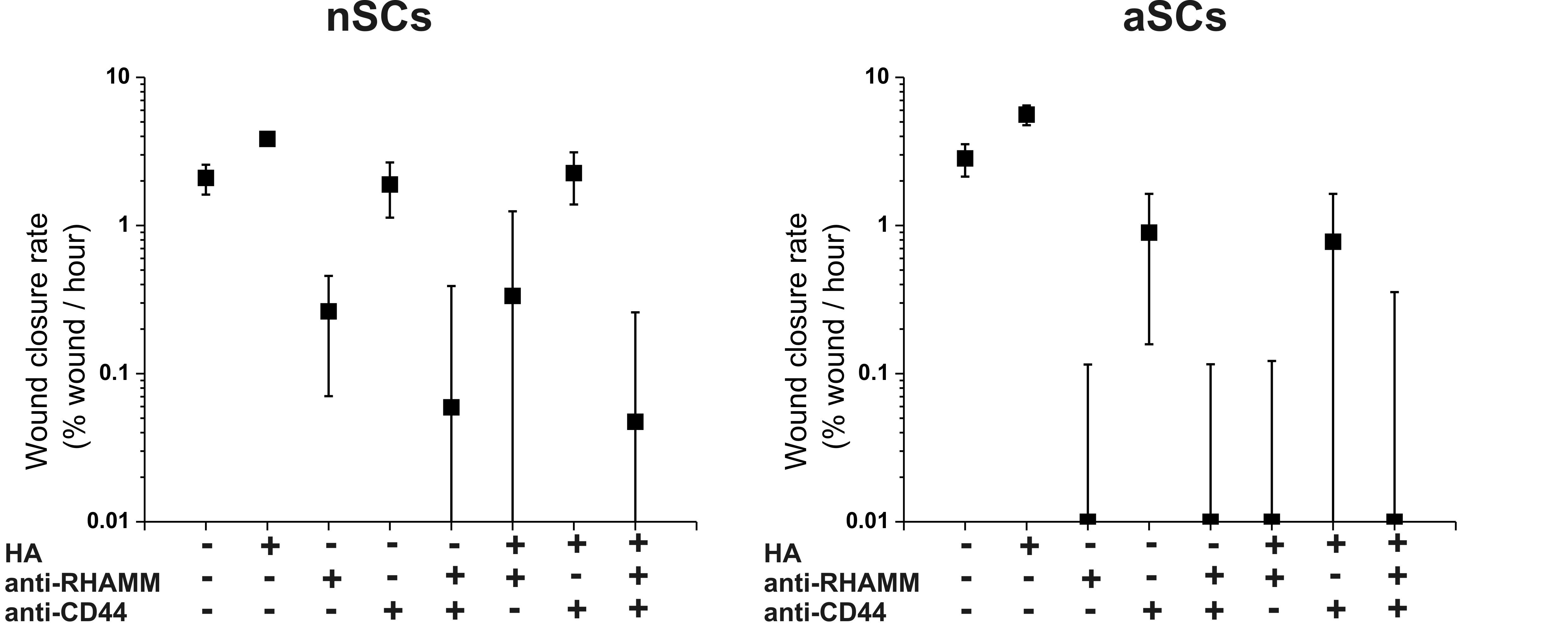


**Figure S6.** Wound closure rate for neonatal (left) and adult Schwann cells (right) as a function of the presence of HA or of antibodies for RHAMM or CD44.

**Table S1** **Sequences of primers for the RT-PCR studies**

|  | Forward primer (5’→3’) | Reverse primer (5’→3’) |
| --- | --- | --- |
| RHAMM | **5’-TAGATATTGCCCAGTTAGAAG-3’** | **5’􏰃-TGAGCAGCAATATGTTTCTCCAGTTC- 3’** |
| CD44 | **5’-TCATGTTAGAGCATCCGTGC-3’** | **5’-GGGTTGTACATCATGCCTCC-3’** |
| β-Actin | **5’-ACTATCGGCAATGAGCGGTTC-3’** | **5’-AGAGCCACCAATCCACACAGA-3’** |

[1] C. Wang, J. Entwistle, G.P. Hou, Q.A. Li, E.A. Turley, The characterization of a human RHAMM cDNA: Conservation of the hyaluronan-binding domains, Gene 174(2) (1996) 299-306.

[2] C. Fieber, R. Plug, J. Sleeman, P. Dall, H. Ponta, M. Hofmann, Characterisation of the murine gene encoding the intracellular hyaluronan receptor IHABP (RHAMM), Gene 226(1) (1999) 41-50.

[3] M. Hofmann, C. Fieber, V. Assmann, M. Gottlicher, J. Sleeman, R. Plug, N. Howells, O. von Stein, H. Ponta, P. Herrlich, Identification of IHABP, a 95kDa intracellular hyaluronate binding protein, Journal of Cell Science 111 (1998) 1673-1684.
